# Sensory Processing and Emotion Regulation in Adults with ADHD and Their Siblings: Preliminary Evidence for a Potential Endophenotype

**DOI:** 10.1192/j.eurpsy.2026.10202

**Published:** 2026-07-31

**Authors:** M. N. Bayram, B. Sağlam, A. Sakallı Kani

**Affiliations:** 1 Department of Psychiatry, Marmara University; 2 Department of Psychology, İstanbul Ticaret University, İstanbul, Türkiye

## Abstract

**Introduction:**

Sensory and emotional dimensions appear to play a central role in ADHD, beyond core symptoms. While evidence in adults is limited, little is known about whether such traits are also observed in unaffected siblings, which could indicate familial or genetic vulnerability.

**Objectives:**

To examine sensory processing profiles and emotion regulation difficulties in adults with ADHD, their siblings, and healthy controls, and to investigate the predictive role of sensory processing in emotion regulation.

**Methods:**

A total of 190 participants were recruited: 80 adults with ADHD, 35 unaffected siblings, and 75 non-ADHD controls. All participants completed the Adult/Adolescent Sensory Profile (AASP) and the Difficulties in Emotion Regulation Scale (DERS). Group comparisons were conducted using one-way ANOVA with post-hoc tests. Pearson correlations and multiple linear regression analyses were performed to examine associations between sensory processing dimensions and emotion regulation difficulties.

**Results:**

Adults with ADHD reported significantly higher scores in emotion dysregulation (DERS total) compared to controls (p < .001), with siblings showing intermediate levels. Group differences were most pronounced in clarity, impulsivity, goals, and strategies subscales.

In terms of sensory processing (AASP), adults with ADHD scored higher in low registration, sensory sensitivity, and sensory avoidance than controls (all p < .01). Siblings displayed intermediate scores, particularly for low registration.

Correlation analyses revealed strong positive associations between low registration, sensory sensitivity, and sensory avoidance with emotion dysregulation (r = .40–.63, p < .001). Multiple regression analysis indicated that low registration was the strongest predictor of overall emotion dysregulation (β = .48, p < .001).

**Conclusions:**

This study demonstrates that adults with ADHD exhibit distinct sensory processing patterns and marked emotion regulation difficulties compared to controls, with siblings showing intermediate profiles. These findings suggest that alterations in low registration and related sensory dimensions may contribute to emotion dysregulation in ADHD. The presence of similar patterns in unaffected siblings raises the possibility that these traits represent a potential endophenotype of ADHD. Further research is needed to confirm these preliminary results and explore their clinical implications.

**Disclosure of Interest:**

None Declared

